# Caracterização Genética e Clínica de uma Coorte de Pacientes com Cardiomiopatia Hipertrófica no Sul do Brasil

**DOI:** 10.36660/abc.20250420

**Published:** 2026-03-04

**Authors:** Thais Beuren, Fernando Scolari, Fernanda Sperb-Ludwig, Filipe Ferrari, Arthur Rossi, Rafael Zawislak, Peter van Tintelen, Ricardo Stein

**Affiliations:** 1 Universidade Federal do Rio Grande do Sul Porto Alegre RS Brasil Universidade Federal do Rio Grande do Sul, Porto Alegre, RS - Brasil; 2 Hospital Moinhos de Vento Porto Alegre RS Brasil Hospital Moinhos de Vento, Porto Alegre, RS - Brasil; 3 Hospital de Clínicas de Porto Alegre Porto Alegre RS Brasil Hospital de Clínicas de Porto Alegre, Porto Alegre, RS - Brasil; 4 Pontifícia Universidade Católica do Rio Grande do Sul Porto Alegre RS Brasil Pontifícia Universidade Católica do Rio Grande do Sul, Porto Alegre, RS - Brasil; 5 University Medical Center Utrecht Utrecht University Utrecht Holanda University Medical Center Utrecht, Utrecht University, Utrecht - Holanda

**Keywords:** Cardiomiopatia Hipertrófica, Teste Genético, Sequenciamento de Nucleotídeos em Larga Escala, Brasil

## Abstract

**Fundamento:**

A cardiomiopatia hipertrófica (CMH) é a doença cardíaca monogênica mais comum, caracterizada por heterogeneidade genética e fenotípica. Embora amplamente estudada em populações norte-americanas e europeias, os dados provenientes do Brasil permanecem limitados.

**Objetivos:**

Caracterizar os perfis genéticos e clínicos de uma coorte sul-brasileira de pacientes com CMH e seus familiares utilizando sequenciamento em paralelo em larga escala.

**Métodos:**

Neste estudo observacional, pacientes com CMH e seus familiares de primeiro grau foram recrutados em ambulatórios de cardiologia. Dados clínicos e de imagem foram coletados, e a análise genética utilizou um painel de 100 genes. A patogenicidade das variantes foi avaliada de acordo com os critérios do *American College of Medical Genetics and Genomics* , e as análises estatísticas foram realizadas utilizando o software R.

**Resultados:**

Oitenta indivíduos foram incluídos na análise final (idade média: 49,2 ± 18,5); 60% do sexo masculino; 40 casos-índice e 40 familiares afetados). MYH7 e MYBPC3 foram os genes mais frequentemente relacionados, com variantes Patogênicas/Provavelmente Patogênicas (P/PP) identificadas em 33% e 16% dos participantes, respectivamente. Nenhuma variante patogênica em TNNT2 foi detectada. Noventa por cento dos participantes apresentaram alguma variante identificada (incluindo variante de significado incerto), sendo que 68% carregavam variantes P/PP. Portadores de MYH7 exibiram maior proporção de obstrução do trato de saída do ventrículo esquerdo, enquanto portadores de MYBPC3 apresentaram maior proporção de eventos arrítmicos e diagnóstico mais precoce; entretanto, essas diferenças não atingiram significância estatística e devem ser interpretadas como exploratórias. As comparações clínicas revelaram diferenças regionais, sugerindo o potencial impacto da diversidade genética na apresentação da CMH nesta região do Brasil.

**Conclusões:**

Este estudo oferece a primeira caracterização genética e clínica detalhada de uma coorte brasileira de pacientes portadores de CMH utilizando sequenciamento em paralelo em larga escala. Nossos achados ressaltam a importância do teste genético para o diagnóstico, a estratificação de risco e o manejo.

## Graphical abstract

**Figura Central:**
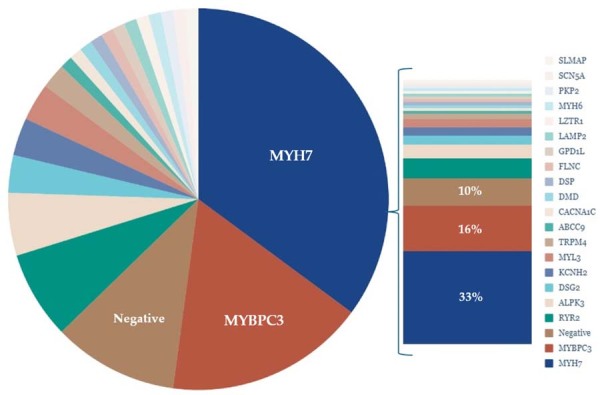
Caracterização Genética e Clínica de uma Coorte de Pacientes com Cardiomiopatia Hipertrófica no Sul do Brasil

Frequências dos genes afetados identificados por sequenciamento em paralelo em larga escala.

## Introdução

A Cardiomiopatia Hipertrófica (CMH) é a doença cardíaca monogênica mais comum, com uma prevalência de aproximadamente 1:250 a 1:500 indivíduos.^1^ É causada principalmente por variantes patogênicas em genes sarcoméricos e relacionados ao sarcômero. A condição é caracterizada por hipertrofia ventricular esquerda não relacionada a condições de sobrecarga anormal e pode levar a complicações como Obstrução da Via de Saída do Ventrículo Esquerdo (OVSVE), Morte Súbita Cardíaca (MSC) e insuficiência cardíaca.^2 - 4^

A expressão da doença é altamente variável, tornando desafiadora a previsão dos desfechos clínicos, mesmo entre indivíduos genótipo-positivos.^5 , 6^ Certas variantes genéticas têm sido associadas a diferentes prognósticos: variantes do gene MYH7 estão relacionadas a piores desfechos, enquanto variantes do MYBPC3 tendem a apresentar início tardio e penetrância incompleta.^7 - 10^ Embora a predição de risco específica por genótipo permaneça controversa, o status genótipo-positivo tem sido associado a início mais precoce da doença, maior disfunção ventricular e aumento da mortalidade cardiovascular.^11 - 15^ Nesse sentido, as diretrizes mais recentes da AHA/ACC incluem o status genótipo-positivo como um fator de risco definitivo para MSC em pacientes pediátricos, com evidências crescentes também em adultos.^6^

Apesar da reconhecida importância da genética para o diagnóstico, avaliação de risco e aconselhamento familiar,^2 , 16^ a avaliação genética permanece subutilizada no Brasil, e os dados específicos da população são limitados. Estudos brasileiros anteriores relataram o MYH7 como o gene mutado mais prevalente, seguido por MYBPC3 e TNNT2,^17 - 19^ uma distribuição consistente, embora não idêntica, à observada em coortes norte-americanas e europeias.^10 , 20^

Este estudo tem como objetivo caracterizar os perfis genéticos e clínicos de pacientes com CMH e de seus familiares em um centro terciário no sul do Brasil, utilizando sequenciamento paralelo em larga escala.

## Métodos

### Declaração de ética

O protocolo do estudo foi aprovado pelo Comitê de Ética Institucional por meio da Plataforma Brasil (CAAE: 554413421.4.1001.5327).

### Participantes e desenho do estudo

O estudo utilizou um desenho observacional, transversal. Os participantes foram recrutados nos ambulatórios de cardiologia do Hospital de Clínicas de Porto Alegre (HCPA), Brasil. Os indivíduos elegíveis incluíram pacientes diagnosticados com CMH e seus familiares de primeiro grau.

Para os pacientes-índice, os critérios de inclusão exigiam diagnóstico confirmado de CMH, definido como espessura da parede ventricular esquerda ≥ 15 mm não explicada por condições anormais de sobrecarga. Para os familiares portadores de uma variante Patogênica (P) ou Provavelmente Patogênica (PP), a inclusão no estudo requeria genopositividade. Entre os familiares incluídos, uma espessura da parede ventricular esquerda ≥ 13 mm foi utilizada para classificá-los como fenótipo-positivo (CMH), enquanto portadores assintomáticos com espessura < 13 mm foram considerados fenótipo-negativo. Os participantes precisavam ter 18 anos ou mais no momento da inclusão. Os critérios de exclusão incluíram a presença de fenótipos que mimetizam a CMH, ausência de evidências clínicas e genéticas de CMH e idade inferior a 18 anos. Todos os participantes receberam informações detalhadas sobre o estudo e forneceram consentimento informado por escrito antes da inclusão. O aconselhamento genético foi oferecido a todos os participantes, tanto antes quanto após a realização dos testes.

### Base de Dados

As variáveis coletadas incluíram data de nascimento, sexo e idade ao diagnóstico de CMH. Foram obtidos dados de histórico familiar, incluindo histórico de CMH e MSC, definida como pelo menos um parente de primeiro ou segundo grau que tenha apresentado morte súbita inexplicada ou parada cardíaca súbita. Variáveis adicionais incluíram episódios de MSC abortada e a presença de arritmias ventriculares, como taquicardia ventricular sustentada e não sustentada ou fibrilação ventricular. Essas arritmias foram identificadas por meio de monitoramento Holter de 24 horas, eletrocardiograma (ECG) de repouso de 12 derivações ou teste de esforço.

Outros dados coletados incluíram tratamento farmacológico basal, comorbidades, implante de dispositivos cardíacos, presença de fibrilação atrial (detectada por Holter de 24 horas ou ECG de repouso de 12 derivações) e status de mortalidade. Medidas eletrocardiográficas também foram registradas. Os parâmetros ecocardiográficos incluíram tamanho do Átrio Esquerdo (AE), Fração de Ejeção do Ventrículo Esquerdo (FEVE), Diâmetro Diastólico Final do Ventrículo Esquerdo (DFVE) e Diâmetro Sistólico Final do Ventrículo Esquerdo (DSVE), espessura da parede ventricular esquerda, presença de OVSVE e gradiente máximo do trato de saída. Os parâmetros de ressonância magnética cardíaca incluíram FEVE, Volume Diastólico Final do Ventrículo Esquerdo (DFVE) e Volume Sistólico Final do Ventrículo Esquerdo (SFVE), volume atrial esquerdo e presença de realce tardio pelo gadolínio.

### Sequenciamento genético

O DNA genômico foi extraído de amostras de saliva, e o teste genético foi realizado utilizando um painel comercial abrangente de cardiomiopatias contendo 100 genes: *ABCC9 ACADVL ACTC1 ACTN2 AGL ALMS1 ALPK3 BAG3 BRAF CACNA1C CACNA1D CALM1 CALM2 CALM3 CASQ2 CBL CDH2 CPT2 CRYAB CSRP3 DES DMD DNAJC19 DOLK DSC2 DSG2 DSP ELAC2 EMD EYA4 FHL1 FKRP FKTN FLNC, GAA, GATA4, GATA5, GJA5, GLA, HCN4, HRAS, JUP, KCNE1, KCNH2, KCNJ2, KCNQ1, KRAS, LAMP2, LMNA, LZTR1, MAP2K1, MAP2K2, MRAS, MTO1, MYBPC3, MYH6, MYH7, MYL2, MYL3, MYL4, MYLK3, NF1, NKX2-5, NRAS, PCCA, PCCB, PKP2, PLN, PPA2, PPCS, PPP1CB, PRKAG2, PTPN11, RAF1, RASA1, RBM20, RIT1, RYR2, SCN5A, SDHA, SGCD, SHOC2, SLC22A5, SOS1, SOS2, SPRED1, TAZ, TBX20, TCAP, TMEM43, TMEM70, TNNC1, TNNI3, TNNI3K, TNNT2, TPM1, TRDN, TRPM4, TTN, TTR, VCL* . O sequenciamento completo dos genes foi realizado utilizando tecnologia de sequenciamento paralelo em larga escala.

### Análise estatística

Os dados do estudo foram coletados e gerenciados utilizando o REDCap (Research Electronic Data Capture), uma plataforma web segura e confidencial.^21^ A análise estatística foi realizada com o software R, versão 4.3.0. As variáveis categóricas foram analisadas por meio do teste do qui-quadrado. Para variáveis contínuas com distribuição normal, foram utilizados testes t independentes, enquanto variáveis sem distribuição normal foram analisadas pelo teste de postos sinalizados de Wilcoxon. Um nível de significância de 5% (p < 0,05) foi considerado para todas as análises.

## Resultados

### Descrição da população geral

**Figura 1:**
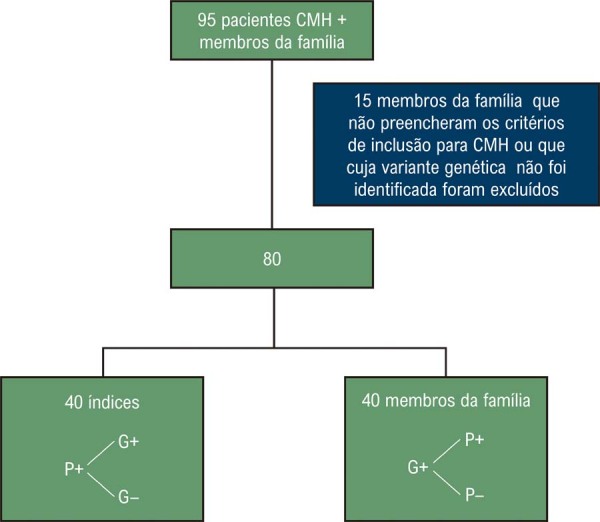
– Fluxograma do processo de seleção. Índice: primeiro caso identificado na família; G+: genótipo positivo; G-: genótipo negativo; P+: fenótipo positivo; P-: fenótipo negativo.

Um total de 95 indivíduos, incluindo pacientes afetados e seus familiares, foram inicialmente triados. Após o teste genético, familiares sem diagnóstico clínico de CMH ou sem variante patogênica ou provavelmente patogênica identificada foram excluídos (n=15). Assim, 80 indivíduos foram incluídos na análise final: 40 pacientes-índice não relacionados e 40 familiares afetados ou portadores de variantes ( figura 1 ). Desses, 60% eram homens (n=48), 50% eram pacientes-índice (n=40) e 50% eram familiares (n=40). Entre os participantes, 68% tinham diagnóstico de CMH (n=54), enquanto 32% eram portadores de variantes (n=26). A média de idade foi de 49,2 anos (DP: 18,5). Histórico familiar de CMH foi observado em 32% dos pacientes-índice (n=13).

A mediana da espessura do septo ventricular esquerdo foi de 18,5 mm (IIQ: 15,0 – 21,0), a espessura da parede posterior foi de 10 mm (IIQ: 9,0 – 12,0) e a FEVE foi de 67% (IIQ: 63,2 – 72,0). OVSVE esteve presente em 33% dos participantes, e arritmias ventriculares foram observadas em 15%. Um resumo das características clínicas e demográficas é apresentado na Tabela 1 .

**Tabela 1:** – Características basais dos pacientes

Características	Total
**Gênero - n, (%)**	
Feminino	32 (40)
Masculino	48 (60)
**Idade (anos)**	
Mínima	18
Máxima	86
Média (DP)	49,2 ± 18,5
**História familiar de CMH (pacientes índices n=40) - no, (%)**	
Sim	13 (32,5)
Não	27 (67,5)
**História familiar de MSC - no, (%)**	
Sim	19 (25,6)
Não	55 (74,4)
**Síncope - no, (%)**	
Sim	13 (17,3)
Não	62 (82,7)
**Arritmia ventricular - no, (%)**	
Sim	12 (15)
Não	68 (85)
**Comorbidades - no, (%)**	
Hipertensão	20 (25,9)
Diabetes	10 (12,9)
Doença renal crônica	3 (4)
Doença arterial coronariana	7 (9,3)
Cardiomiopatia dilatada	3 (4)
Implante de cardiodesfibrilador implantável	10 (13,3)
Fibrilação atrial	11 (14,6)

CMH: cardiomiopatia hipertrófica; MSC: morte súbita cardíaca.

### Perfil genético

**Figura 2:**
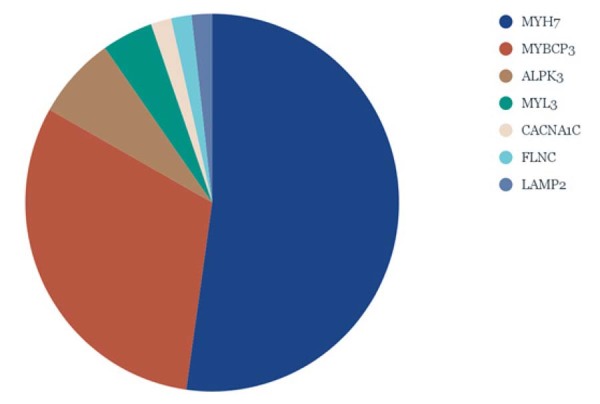
– Distribuição dos genes afetados fortemente ou moderadamente relacionados à cardiomiopatia hipertrófica.

Entre todos os genes sequenciados, variantes P, PP e VUS foram identificadas em 72 indivíduos (90%). Considerando apenas as variantes P e PP, 54 indivíduos (68%) foram classificados como tendo teste genético positivo. Para a caracterização inicial, todas as variantes identificadas estão apresentadas na Figura Central . A Figura 2 mostra a distribuição das variantes P/PP em genes fortemente ou moderadamente associados à CMH.^22^ Os dois genes mais frequentemente encontrados na CMH são destacados: MYH7 (33%) e MYBPC3 (16%). Além disso, 10% da amostra não apresentou variantes identificadas.

Seis indivíduos exibiram variantes relacionadas à CMH em mais de um gene, sem que fosse identificado um padrão específico ( Tabela 2 ).

**Tabela 2:** – Indivíduos com múltiplas variantes

ID	Genes	Variante	Patogenicidade	Afetado	História familiar de CMH	Idade no diagnóstico	Parede do VE	OVSVE	Arritmia ventricular esquerda
1	MYH7	c,2605C>T	Patogênica	Sim	Pai	46	14mm	No	No
	ALPK3	c,1621C>T	Provavelmente patogênica						
	TRPM4	c,1934G>A	VUS						
2	MYH7	c,1987C>T	Patogênica	Sim	Índice	23	17mm	No	No
	LZTR1	c,1957C>T	Provavelmente patogênica						
3	MYBPC3	c,2047T>A	VUS* (~LP)	Sim	Índice	47	17mm	No	Yes (NSVT)
	MYBPC3	c,3107G>A	VUS						
	RYR2	c,4273A>G	VUS						
4	ALPK3	c,2649 2650 delinsT	Patogênica	Não	Pai	-	10mm	-	No
	TRPM4	c,1934G>A	VUS						
5	ALPK3	c,2649 2650 delinsT	Patogênica	Não	Avô	-	9mm	-	No
	DMD	c,5615C>T	VUS						
	GPD1L	c,1009G>C	VUS						
6	MYH7	c,2605C>T	Patogênica	Sim	Índice	60	20mm	Yes	No
	ALPK3	c,1621C>T	Provavelmente patogênica						
	MYBPC3	c,292+138G>A	VUS						

CMH: cardiomiopatia hipertrófica; OVSVE: obstrução da via de saída do ventrículo esquerdo, MSC: morte súbita cardíaca; VUS: variante de significado incerto; * VUS com escore elevado na classificação do American College of Medical Genetics and Genomics e com potencial para reclassificação (Tavtigian et al.^39^).

Para ilustrar a importância de uma avaliação genética e clínica abrangente, apresentamos em detalhe um caso familiar intrigante (ver Material Suplementar). A patogenicidade das variantes foi classificada de acordo com os critérios do *American College of Medical Genetics and Genomics* (ACMG).^2 3^ A distribuição da classificação de patogenicidade das variantes foi a seguinte: 1) patogênicas (44%), 2) provavelmente patogênicas (34%) e 3) Variantes de Significado Incerto (VUS, do inglês *Variant of Uncertain Significance* , 22%). Todas as variantes identificadas em MYH7 e MYBPC3 foram do tipo *missense* , exceto uma variante intrônica em MYH7. Uma descrição detalhada de todas as variantes pode ser encontrada na Tabela 3 .

**Tabela 3:** – Descrição das variantes

Gene	Transcrito	Variante	Classificação	Frequência na população (GnomAD v.4.1.0)
ABCC9	NM_020297,4	c,3559G >T	VUS*	1,86e-6
ALPK3	NM_020778,5	c,1621C>T	PP	Não identificado
ALPK3	NM_020778,5	c,2649 2650 delinsT	P	Não identificado
CACNA1C	NM_000719,7	c,1342 G>A	VUS* (~PP)	5,59e-6
DMD	NM_004006,3	c,5615C>T	VUS	8,28e-7
DSG2	NM_001943,4	c,2505-2506insG	VUS	
DSP	NM_004415,4	c,1742C>T	VUS	Não identificado
FLNC	NM_001458,5	c,3964+1G>T	VUS*	Não identificado
GPD1L	NM_015141,4	c,1009G>C	VUS	1,86e-6
KCNH2	NM_000238,4	c,1898A>C	PP	Não identificado
KCNH2	NM_000238,4	c,526C>T	PP	3,27e-4
KCNH2	NM_000238,4	c,2863C>G	VUS	5,80e-5
LAMP2	NM_001122606,1	c,1234T>C	VUS	Não identificado
LZTR1	NM_006767,4	c,1957C>T	PP	2,48e-6
MYBPC3	NM_000256,3	c,1484G>A	P	2,42e-5
MYBPC3	NM_000256,3	c,1633C>A	VUS*	2,17e-5
MYBPC3	NM_000256,3	c,2047T>A	VUS*	Não identificado
MYBPC3	NM_000256,3	c,292+138G>A	VUS	
MYBPC3	NM_000256,3	c,3107G>A	VUS	7,09e-5
MYBPC3	NM_000256,3	c,3065G>C	VUS*	5,21e-5
MYBPC3	NM_000256,3	c,1505G>A	P	6,82e-6
MYH6	NM_002471,4	c,3010G>T	VUS	1,10e-3
MYH7	NM_000257,3	c,2737G>T	LP	
MYH7	NM_000257,3	c,4508G>A	P	
MYH7	NM_000257,4	c,1987C>T	P	1,86e-6
MYH7	NM_000257,4	c,2572C>G	P	2,48e-6
MYH7	NM_000257,4	c,2605C>T	P	1,86e-6
MYH7	NM_000257,4	c,3972+63A>CA	VUS	
MYH7	NM_000257,4	c,4402G>A	P	Não identificado
MYH7	NM_000257,4	c,1063G>A	P	4,34e-6
MYH7	NM_000257,4	c,2389G>A	P	2,85e-5
MYH7	NM_000257,4	c,788T>C	P	3,72e-6
MYL3	NM_000258,3	c,170C>G	P	5,14e-5
MYL3	NM_000258,3	c,187C>T	VUS	1,30e-5
PKP2	NM_001005242,3	c,2464A>G	VUS	3,97e-5
RYR2	NM_001035,3	c,1249C>G	VUS*	1,36e-5
RYR2	NM_001035,3	c,147G>T	VUS*	8,18e-6
RYR2	NM_001035,3	c,593A>T	VUS	1,86e-6
SCN5A	NM_000335,5	c,3612C>A	VUS	Not found
SLMAP	NM_001377540,1	c,2080C>T	VUS	6,20e-7
TRPM4	NM_017636,4	c,1934G>A	VUS	1,05e-5

PP: provavelmente patogênico; P: patogênico; VUS: variante de significado incerto; * VUS com escore elevado na classificação do American College of Medical Genetics and Genomics e potencial para reclassificação (Tavtigian et al.^39^).

### Caracterização clínico-genética de indivíduos fenótipo-positivos

Para compreender melhor como o histórico genético influencia o fenótipo da CMH, descrevemos e comparamos as características clínicas de pacientes com CMH portadores de diferentes genes afetados ( Tabela 4 ). Esta análise concentrou-se exclusivamente em genes com correlação bem estabelecida com a CMH, especificamente MYH7 e MYBPC3, com base no tamanho da amostra disponível ( Tabela 5 ). Nossa comparação entre MYBPC3 e MYH7 revelou diferenças relevantes nas frequências, embora estas não tenham alcançado significância estatística. Pacientes com variantes em MYH7 apresentaram maior frequência de obstrução do trato de saída do ventrículo esquerdo (OVSVE) em comparação com aqueles com variantes em MYBPC3 (35% vs. 18%; p = 0,3). Por outro lado, pacientes com variantes em MYBPC3 demonstraram um fenótipo mais arritmogênico, caracterizado por maior prevalência de arritmias ventriculares (33% vs. 17%; p = 0,3), maior taxa de implante de cardiodesfibrilador implantável (CDI) (27% vs. 4%; p = 0,09), maior frequência de fibrose ventricular identificada por realce tardio pelo gadolínio na ressonância magnética cardíaca (62% vs. 38%; p = 0,5) e maior frequência de histórico familiar de morte súbita (45% vs. 35%; p = 0,4). Além disso, a mediana da idade ao diagnóstico dos indivíduos afetados por variantes em MYBPC3 foi menor (37 anos versus 46 anos; p = 0,07). Outras características clínicas estão detalhadas na Tabela 5 .

**Tabela 4:** – Características clínicas dos indivíduos genótipo-fenótipo positivos

Característica	Total (N=53)	MYH7 (N=24)	MYBPC3 (N=12)	MYL3 (N=2)	ALPK3 (N=1)
**Gênero - no, (%)**					
Masculino	33 (66)	16 (67)	7 (58)	2 (100)	1 (100)
Feminino	18 (34)	8 (33)	5 (42)	0 (0)	0 (0)
**Idade - anos**					
Min	18	18	35	18	50
Max	86	86	73	32	50
Média (DP)	54,9 ± 16,9	58,2 ± 17,3	51,1 ± 13,5	21,5 ± 14,8	50
**Idade no diagnóstico de CMH - anos**					
Min	10	20	18	10	44
Max	73	73	60	29	44
Mediana (IIQ)	44 (30,5–53,0)	46,5 (43,2–60,5)	37,5 (21,0–46,0)	19,5 (14,7–24,2)	44 (44,0–44,0)
**Fração de ejeção do VE - %**					
Min	30	30	63	75	68
Max	79	78	79	75	68
Mediana (IIQ)	67 (63,2–72,0)	68,5 (64,0–72,0)	67 (64,5–70,5)	75 (75,0–75,0)	68 (68,0–68,0)
**Septo - mm**					
Min	7	10	10	13	14
Max	29	29	25	13	14
Mediana (IIQ)	18,5 (15,0–21,0)	17 (13,2–21,0)	19 (17,5–21,0)	13 (13,0–13,0)	14 (14,0–14,0)
**Parede posterior - mm**					
Min	7	8	8	9	9
Max	18	15	12	9	9
Mediana (IIQ)	10 (9,0–12,0)	10 (9,0–12,0)	10 (9,0–10,5)	9 (9,0–9,0)	9 (9,0–9,0)
**OVSVE - n, (%)**					
Não	32 (67)	13 (65)	9 (82)	1 (100)	1 (100)
Sim	16 (33)	7 (35)	2 (18)	0 (0)	0 (0)
**Arritmia ventricular - no, (%)**					
Não	41 (77)	20 (83)	8 (67)	2 (100)	1 (100)
Sim	12 (23)	4 (17)	4 (33)	0 (0)	0 (0)
**Fibrilação atrial - no, (%)**					
Não	40 (78)	14 (61)	9 (82)	1 (100)	1 (100)
Sim	11 (22)	9 (39)	2 (18)	0 (0)	0 (0)
**Realce tardio pelo gadolínio - n, (%)**					
Não	21 (60)	8 (62)	3 (37,5)	0 (0)	1 (100)
Sim	14 (40)	5 (38)	5 (62,5)	1 (100)	0 (0)
**Tipo de Arritmia Ventricular - n, (%)**					
TVNS	11 (21)	4 (17)	4 (33)	0 (0)	0 (0)
TVS	2 (4)	2 (8)	0 (0)	0 (0)	0 (0)
FV	1 (2)	0 (0)	0 (0)	0 (0)	0 (0)

CMH: cardiomiopatia hipertrófica; IIQ: intervalo interquartil; FV: fibrilação ventricular TVNS: taquicardia ventricular não sustentada; TVS: taquicardia ventricular sustentada; VE: ventrículo esquerdo.

**Tabela 5:** – Características Clínicas dos Indivíduos com Alterações em MYH7 e MYBPC3

Característica	MYH7 (N=24)	MYBPC3 (N=12)	Valor p
**Gênero - no, (%)**			
Masculino	16 (67)	7 (58)	0,6003
Feminino	8 (33)	5 (42)	
**Idade - anos**			
Min	18	35	
Max	86	73	
Média (DP)	58,21 ± 17,33	51,17 ± 13,54	0,1925
**Idade no diagnóstico de CMH - anos**			
Min	20	18	
Max	73	60	
Mediana (IIQ)	46,5 (43,2–60,5)	37,5 (21,0–46,0)	0,0753
**Fração de ejeção do VE - %**			
Min	30	63	
Max	78	79	
Mediana (IIQ)	68,5 (64,0–72,0)	67 (64,5–70,5)	1,000
**Septo - mm**			
Min	10	10	
Max	29	25	
Mediana (IIQ)	17 (13,2–21,0)	19 (17,5–21,0)	0,319
**Parede posterior - mm**			
Min	8	8	
Max	15	12	
Mediana (IIQ)	10 (9,0–12,0)	10 (9,0–10,5)	0,4272
**OVSVE - no, (%)**			
Não	13 (65)	9 (82)	0,3069
Sim	7 (35)	2 (18)	
**Arritmia ventricular - no, (%)**			
Não	20 (83)	8 (67)	0,3088
Sim	4 (17)	4 (33)	
**Fibrilação atrial - no, (%)**			
Não	14 (61)	9 (82)	0,2072
Sim	9 (39)	2 (18)	
**Realce tardio pelo gadolínio - no, (%)**			
Não	8 (62)	3 (37,5)	0,5344
Sim	5 (38)	5 (62,5)	
**Tipo de Arritmia Ventricular - no, (%)**			
TVNS	4 (17)	4 (33)	0,3088
TVS	2 (8)	0 (0)	0,8878
FV	0 (0)	0 (0)	NA
**Dispositivos cardíacos - no, (%)**			
Desfibrilador Cardíaco Implantável	4 (17)	3 (27)	0,094
Marcapasso Cardíaco Artificial	4 (17)	0 (0)	
DCI + MPA	2 (9)	0 (0)	
Terapia de ressincronização cardíaca	0 (0)	0 (0)	
TRC + ICD	0 (0)	0 (0)	
Nenhum	16 (70)	8 (73)	
**História familiar de MSC - no, (%)**			
Não	3 (14)	1 (9)	0,567
Sim	8 (35)	5 (45)	NA
**MSC abortada - no, (%)**			
Não	22 (96)	12 (100)	1,000
Sim	1 (4)	0 (0)	

CMH: cardiomiopatia hipertrófica; DP: desvio padrão; FB: fibrilação ventricular; IIQ: intervalo interquartil; DCI: desfibrilador cardíaco implantável; MCA: marcapasso cardíaco artificial; OVSVE: obstrução da via de saída do ventrículo esquerdo; TVNS: taquicardia ventricular não sustentada; TRC: terapia de ressincronização cardíaca; TVS: taquicardia ventricular sustentada TRC: terapia de ressincronização cardíaca,

### Genótipo-positivo versus Genótipo-negativo em indivíduos afetados

Entre os indivíduos diagnosticados com CMH, comparamos as características daqueles com variantes genéticas identificadas (fenótipo-positivo/genótipo-positivo) com os que apresentaram teste genético negativo (fenótipo-positivo/genótipo-negativo). A idade média ao diagnóstico de CMH foi de 44 anos nos indivíduos genótipo-positivo e de 50 anos nos pacientes genótipo-negativo (p = 0,86). A mediana da espessura do septo ventricular esquerdo foi de 17,5 mm nos indivíduos genótipo-positivo, em comparação com 21 mm nos indivíduos genótipo-negativo (p = 0,18). Arritmias ventriculares estiveram presentes em 25% dos indivíduos genótipo-positivo e em 12% dos genótipo-negativo (p = 0,77), fibrilação atrial em 23,3% e 12,5% (p = 0,83), e realce tardio pelo gadolínio em 48,2% e 0% (p = 0,08), respectivamente, nos grupos genótipo-positivo e genótipo-negativo. Histórico familiar de MSC foi relatado em 38% do grupo genótipo-positivo e em 25% do grupo genótipo-negativo (p = 0,76). Os detalhes estão resumidos na Tabela 6 .

**Tabela 6:** – Características Clínicas dos Indivíduos Genótipo + e Genótipo - Afetados

Característica	Negativo (N=8)	Positivo (N=45)	Valor p
**Sexo – n (%)**			0,8605
Masculino	6 (75)	29 (64)	
Feminino	2 (25)	16 (36)	
**Idade – anos**			
Mínimo	40	11	
Máximo	80	86	
Média (DP)	58,12 ± 15,34	54,40 ± 17,29	0,5483
**Idade ao diagnóstico de CMH – anos**			
Mínimo	50	10	
Máximo	50	73	
**Mediana (IIQ)**	50 (50,0–50,0)	44 (29,75–53,50)	0,5631
Fração de ejeção – %			
Mínimo	57	30	
Máximo	71	79	
**Mediana (IIQ)**	66 (62,5–67,5)	68 (64,0–72,0)	0,1981
Septum – mm			
Mínimo	7	10	
Máximo	26	29	
**Mediana (IIQ)**	21 (18,7–22,0)	17,4 (14,2–21,0)	0,1884
Parede posterior – mm			
Mínimo	7	8	
Máximo	18	15	
**Mediana (IIQ)**	11,5 (8,7–13,5)	10,4 (9,0–12,0)	0,4752
Obstrução da via de saída do VE – n (%)			0,4936
Não	4 (50)	28 (70)	
Sim	4 (50)	12 (30)	
**Arritmia ventricular – n (%)**			0,7753
Não	7 (88)	34 (75)	
Sim	1 (12)	11 (25)	
**Fibrilação atrial – n (%)**			0,8328
Não	7 (87,5)	33 (77)	
Sim	1 (12,5)	10 (23)	
**Realce tardio por gadolínio – n (%)**			0,082
Não	6 (100)	15 (52)	
Sim	0 (0)	14 (48)	
**Tipo de arritmia ventricular – n (%)**			
TV não sustentada	1 (12,5)	10 (22)	0,8794
TV sustentada	0 (0)	2 (4)	1
Fibrilação ventricular	1 (12,5)	0 (0)	0,3249
**Dispositivos cardíacos – n (%)**			0,582
CDI	2 (25)	6 (14)	
Marcapasso	0 (0)	5 (12)	
CDI + marcapasso	0 (0)	3 (7)	
TRC	0 (0)	0 (0)	
TRC + CDI	0 (0)	0 (0)	
Nenhum	6 (75)	29 (67)	
**História familiar de MSC – n (%)**			0,7601
Não	6 (75)	26 (62)	
Sim	2 (25)	16 (38)	

DP: desvio padrão; FB: fibrilação ventricular; IIQ: intervalo interquartil; DCI: desfibrilador cardíaco implantável; MCA: marcapasso cardíaco artificial; OVSVE: obstrução da via de saída do ventrículo esquerdo; TVNS: taquicardia ventricular não sustentada; TRC: terapia de ressincronização cardíaca; TVS: taquicardia ventricular sustentada TRC: terapia de ressincronização cardíaca

## Discussão

Neste estudo, verificamos que MYH7 e MYBPC3 foram os genes mais prevalentes na coorte de pacientes com CMH do sul do Brasil, com variantes detectadas em 33% e 16% dos indivíduos, respectivamente. Esses achados são consistentes com estudos anteriores, nos quais MYH7 e MYBPC3 foram identificados como os genes mais frequentemente implicados na CMH, respondendo por 30%–50% dos casos.^2 , 3 , 10^ No entanto, nosso estudo revela uma distribuição ligeiramente diferente. De modo geral, a frequência dessas variantes está alinhada ao que tem sido observado em outras populações, embora a proporção de casos sem variantes identificadas continue representando um desafio para o diagnóstico genético da CMH.

Curiosamente, ao contrário da maioria das coortes europeias e americanas, nenhum indivíduo em nosso estudo apresentou variantes em TNNT2.^24^ É importante destacar que cerca de 30 genes já foram implicados na CMH, sendo que 21 deles apresentam evidência moderada de associação gene-doença. Até o momento, mais de 2.000 variantes foram documentadas.^9^ A maioria dessas variantes codifica proteínas sarcoméricas ou está relacionada à estrutura do sarcômero, componentes críticos da maquinaria contrátil cardíaca. Variantes genéticas menos comuns, também associadas à CMH, envolvem genes que codificam proteínas relacionadas ao sarcômero, como ACTN2, ALPK3, CSRP3, FHOD3, JPH2, KLHL24, MT-TI, PLN e TRIM63,^2 2^ que em nossa coorte corresponderam a uma proporção menor, conforme previamente descrito.

As diferenças observadas nas características clínicas entre portadores de variantes em MYH7 e MYBPC3 — como maior frequência de OVSVE em MYH7 e tendências a eventos arrítmicos e diagnóstico mais precoce em MYBPC3 — não alcançaram significância estatística. Portanto, esses achados devem ser interpretados como exploratórios e geradores de hipóteses, em vez de indicativos de relações causais. Além disso, a inclusão de múltiplos membros da mesma família pode introduzir viés, já que fatores genéticos e ambientais compartilhados podem influenciar as tendências observadas. Estudos futuros com coortes maiores são necessários para esclarecer potenciais correlações genótipo-fenótipo.

Ao contrário de muitas coortes previamente descritas, encontramos uma proporção mais elevada de variantes P/PP. Uma meta-análise de Topriceanu et al.^25^ relata uma taxa global de variantes P/PP de aproximadamente 30–40% entre pacientes com CMH. Nossos achados podem refletir viés de seleção devido à disponibilidade limitada de testes genéticos, com preferência por selecionar pacientes com maior probabilidade de um teste positivo (isto é, casos familiares). Além disso, indivíduos com consultas cardiológicas mais frequentes – geralmente aqueles com fenótipos mais graves – podem ter sido mais propensos a realizar testes genéticos.

Este primeiro relato de uma coorte brasileira de CMH com rastreamento genético extensivo tem como objetivo esclarecer os achados genotípicos, incluindo aqueles relacionados a genes menos estabelecidos. Entretanto, não foram identificados padrões específicos, apesar de alguns indivíduos apresentarem múltiplas variantes. A distribuição das variantes pode diferir entre gerações, e permanece incerto como a gravidade da doença é influenciada por uma única variante em comparação com múltiplas variantes. Relatos anteriores sugerem que genes modificadores ou variantes adicionais em genes sarcoméricos podem contribuir para uma expressão fenotípica mais grave da CMH.^14 , 26 - 31^ Em nossa coorte, observamos duas VUS em genes com evidência limitada de associação com CMH, a saber, ABCC9 e RYR2. Além disso, identificamos uma jovem jogadora de futebol que apresentou hipertrofia significativa do ventrículo esquerdo sem preencher os critérios para síndrome de Noonan. Ela carregava uma variante patogênica em MYH7 e uma variante patogênica em LZTR1 (associada à síndrome de Noonan), o que pode representar uma fenocópia. Um exemplo intrigante também é fornecido por uma família na qual quatro indivíduos carregavam variantes patogênicas em ambos os genes MYH7 e ALPK3, conforme detalhado no Material Suplementar.

É importante enfatizar o impacto do diagnóstico das doenças cardíacas genéticas, não apenas nos pacientes, mas também nos familiares em risco. O aconselhamento genético é indispensável, pois demonstrou reduzir a ansiedade, melhorar a percepção de risco, ampliar o conhecimento sobre a doença e promover apoio emocional^31^ Além disso, as discussões de casos em equipes multidisciplinares são inestimáveis e essenciais para o manejo eficaz desses pacientes.

A correlação do perfil genético como a expressão da doença na CMH – seja em termos de fenótipo ou de desfechos adversos – continua sendo um grande desafio. As ferramentas atuais de predição de risco para MSC baseiam-se principalmente em marcadores clínicos estabelecidos, como espessura da parede ventricular esquerda, síncope, disfunção sistólica do ventrículo esquerdo, OVSVE, taquicardia ventricular não sustentada, realce tardio pelo gadolínio e histórico familiar de MSC.^32 , 33^ Estudos anteriores relataram resultados inconsistentes quanto às correlações genótipo-fenótipo na gravidade da doença. Watkins et al.^13^ demonstraram redução da expectativa de vida em pacientes com variantes missense em MYH7, enquanto Jansen et al.^14^ observaram desfechos clínicos mais desfavoráveis associados a mutações em MYH7. Além disso, variantes em MYH7 têm sido associadas a maior risco de fibrilação atrial.^15^ No entanto, evidências emergentes sugerem que certas variantes em MYH7 podem estar correlacionadas a manifestações mais atenuadas da doença. Notavelmente, Marsili et al.^34^ identificaram recentemente início tardio da doença com progressão gradual dos sintomas, particularmente entre mulheres portadoras da variante patogênica MYH7 p.Arg1712Gln. Esses achados contrastantes ressaltam a importância de investigações mais detalhadas sobre as relações genótipo-fenótipo, levando em consideração os efeitos específicos de cada variante.

Enquanto isso, variantes patogênicas em MYBPC3 têm sido associadas a início mais tardio da doença e maiores taxas de penetrância incompleta em comparação com variantes em MYH7.^14^ Por outro lado, certas variantes em TNNT2 (Arg92Trp, Arg92Gln e Ile79Asn) foram associadas a risco aumentado de MSC em famílias afetadas.^27^ Embora as diferenças entre os indivíduos afetados em nossa coorte não tenham alcançado significância estatística, é plausível que pacientes com genótipos positivos apresentem doença mais grave. Importante destacar que, em nosso estudo, portadores de variantes em MYBPC3 mostraram maior proporção de características associadas à MSC – incluindo mais arritmias ventriculares, maiores taxas de implante de cardiodesfibrilador implantável, maior prevalência de histórico familiar de MSC e idade mais precoce ao diagnóstico de CMH.

Por outro lado, observamos maior frequência de OVSVE entre portadores de variantes em MYH7, enquanto a prevalência de insuficiência cardíaca pareceu semelhante entre os grupos. Enquanto mutações em MYH7 interrompem as interações das proteínas sarcoméricas, a patogenicidade de MYBPC3 parece ser impulsionada principalmente pela haploinsuficiência – seja por degradação do mRNA mediada por nonsense ou pela produção de proteínas defeituosas incapazes de se integrar ao sarcômero.^35 , 36^ Um estudo brasileiro de Marsiglia et al. apoia essa hipótese ao identificar maior proporção de variantes truncantes em MYBPC3.^17^ Em nossa análise, todas as variantes de MYBPC3 foram do tipo *missense* ; entretanto, isso não implica necessariamente em um fenótipo mais brando.

Coletivamente, esses achados ressaltam a necessidade de investigação adicional sobre os mecanismos moleculares de patogenicidade do MYBPC3. Apesar de numerosos estudos, ainda não foram estabelecidos preditores genótipo-fenótipo definitivos. A expressão da doença provavelmente varia de acordo com variantes específicas, e não apenas pelo gene em si. Além dos fatores de risco cardiovasculares tradicionais (por exemplo, hipertensão, excesso de peso) que influenciam o desenvolvimento da CMH, variantes genéticas comuns têm demonstrado explicar uma parcela significativa da herdabilidade da doença.^37 , 38^ Assim, a caracterização detalhada de variantes específicas pode ajudar a refinar escores de risco poligênico no futuro. Esforços globais em andamento provavelmente impulsionarão avanços nessa área.

Embora a relação entre genes específicos e a gravidade da doença permaneça controversa, a presença de um genótipo positivo (independentemente do gene) tem sido consistentemente associada a piores desfechos clínicos. Ho et al.^16^ identificaram o teste genético positivo como o melhor preditor independente de eventos adversos em pacientes com CMH. De forma semelhante, Marsiglia et al.^19^ observaram que indivíduos brasileiros com genótipos positivos apresentaram diagnóstico mais precoce de CMH e maior incidência de arritmias ventriculares. Em nosso estudo, não houve diferença entre os grupos, embora tenhamos observado proporções semelhantes: maior frequência de arritmias ventriculares, fibrilação atrial e fibrose ventricular nos pacientes genótipo-positivo, juntamente com diagnóstico mais precoce e maior histórico familiar de MSC. Esses achados reforçam a importância de reconhecer a CMH como uma doença de base genética, independentemente do gene específico envolvido.

Apesar da complexidade inerente da CMH, há evidências substanciais que sustentam o papel central da genética no manejo da doença. O padrão de herança autossômica dominante, com 50% de chance de transmissão, tem importantes implicações para o cuidado médico e para as decisões de vida dos familiares em risco.^1^ No Brasil, assim como em muitos países em desenvolvimento, a genética é frequentemente subutilizada no manejo da CMH devido ao alto custo, ao conhecimento limitado dos médicos e à insuficiente conscientização sobre seu impacto clínico. Isso resulta em um cuidado subótimo aos pacientes. Para melhorar o manejo da CMH, é crucial promover testes genéticos mais abrangentes, utilizando técnicas genômicas avançadas em amostras populacionais maiores. Acreditamos que a defesa da integração da genética na rotina do manejo da CMH poderia, gradualmente, reduzir os custos e ampliar o acesso, melhorando, em última análise, o cuidado aos pacientes com CMH em países em desenvolvimento.

### Limitações

A principal limitação do nosso estudo é o tamanho reduzido da amostra, baseada localmente. Dada a heterogeneidade da CMH, uma coorte maior que inclua indivíduos de diferentes regiões do Brasil proporcionaria uma compreensão mais abrangente das características genéticas da nossa população. Além disso, a inclusão de pacientes provenientes principalmente de um centro terciário pode ter introduzido viés de seleção, já que esses indivíduos têm maior probabilidade de representar um subconjunto mais grave ou especializado de casos de CMH. Isso pode ter influenciado tanto a frequência quanto a classificação das variantes identificadas.

Além disso, a inclusão de membros da família pode ter inflado o rendimento diagnóstico dos testes genéticos. A exclusão de parentes fenótipo-negativos sem variantes patogênicas também limita nossa capacidade de estimar a penetrância e avaliar correlações genótipo-fenótipo entre famílias. Análises formais de agregação familiar e segregação genética não foram realizadas devido ao tamanho limitado da amostra e ao desenho transversal. Além disso, este estudo não avaliou o impacto clínico dos testes genéticos, como possíveis mudanças no manejo dos pacientes ou nas práticas de rastreamento familiar, nem incluiu acompanhamento longitudinal para avaliar desfechos clínicos como morte súbita, hospitalização ou progressão para insuficiência cardíaca. A expansão da amostra para incluir pacientes de centros de atenção primária e secundária poderia ajudar a mitigar esse viés e oferecer uma representação mais precisa da população brasileira com CMH em geral.

## Conclusão

Nesta coorte de pacientes com CMH do sul do Brasil e seus familiares, MYH7 e MYBPC3 foram os genes mais frequentemente mutados, sem variantes patogênicas identificadas em TNNT2. Observamos uma frequência mais elevada de variantes P/PP em comparação com coortes internacionais. A hipótese sobre as associações não significativas das variantes em MYBPC3 com marcadores de gravidade da doença deve ser explorada em estudos futuros. Esses achados sugerem importante heterogeneidade genética e fenotípica dentro desta coorte brasileira de CMH e reforçam o valor clínico do teste genético para estratificação de risco e manejo.

## Material suplementar

Electronic Supplemental Material

## References

[1] Semsarian C, Ingles J, Maron MS, Maron BJ (2015). New Perspectives on the Prevalence of Hypertrophic Cardiomyopathy. J Am Coll Cardiol.

[2] Lopes LR, Ho CY, Elliott PM (2024). Genetics of Hypertrophic Cardiomyopathy: Established and Emerging Implications for Clinical Practice. Eur Heart J.

[3] Ommen SR, Ho CY, Asif IM, Balaji S, Burke MA, Day SM (2024). 2024 AHA/ACC/AMSSM/HRS/PACES/SCMR Guideline for the Management of Hypertrophic Cardiomyopathy: A Report of the American Heart Association/American College of Cardiology Joint Committee on Clinical Practice Guidelines. Circulation.

[4] Elliott PM, Anastasakis A, Borger MA, Borggrefe M, Cecchi F, Charron P (2014). 2014 ESC Guidelines on Diagnosis and Management of Hypertrophic Cardiomyopathy: The Task Force for the Diagnosis and Management of Hypertrophic Cardiomyopathy of the European Society of Cardiology (ESC). Eur Heart J.

[5] Maron BJ, Desai MY, Nishimura RA, Spirito P, Rakowski H, Towbin JA (2022). Diagnosis and Evaluation of Hypertrophic Cardiomyopathy: JACC State-of-the-Art Review. J Am Coll Cardiol.

[6] Ommen SR, Mital S, Burke MA, Day SM, Deswal A, Elliott P (2020). 2020 AHA/ACC Guideline for the Diagnosis and Treatment of Patients with Hypertrophic Cardiomyopathy: Executive Summary: A Report of the American College of Cardiology/American Heart Association Joint Committee on Clinical Practice Guidelines. Circulation.

[7] Claes GR, van Tienen FH, Lindsey P, Krapels IP, Helderman-van den Enden AT, Hoos MB (2016). Hypertrophic Remodelling in Cardiac Regulatory Myosin Light Chain (MYL2) Founder Mutation Carriers. Eur Heart J.

[8] Arbelo E, Protonotarios A, Gimeno JR, Arbustini E, Barriales-Villa R, Basso C (2023). 2023 ESC Guidelines for the Management of Cardiomyopathies. Eur Heart J.

[9] Maron BJ, Maron MS, Semsarian C (2012). Genetics of Hypertrophic Cardiomyopathy after 20 Years: Clinical Perspectives. J Am Coll Cardiol.

[10] Marian AJ, Braunwald E (2017). Hypertrophic Cardiomyopathy: Genetics, Pathogenesis, Clinical Manifestations, Diagnosis, and Therapy. Circ Res.

[11] Roberts R, Sigwart U (2001). New Concepts in Hypertrophic Cardiomyopathies, Part I. Circulation.

[12] Marian AJ, Roberts R (1998). Molecular Genetic Basis of Hypertrophic Cardiomyopathy: Genetic Markers for Sudden Cardiac Death. J Cardiovasc Electrophysiol.

[13] Watkins H, Rosenzweig A, Hwang DS, Levi T, McKenna W, Seidman CE (1992). Characteristics and Prognostic Implications of Myosin Missense Mutations in Familial Hypertrophic Cardiomyopathy. N Engl J Med.

[14] Jansen M, Brouwer R, Hassanzada F, Schoemaker AE, Schmidt AF, Kooijman-Reumerman MD (2024). Penetrance and Prognosis of MYH7 Variant-Associated Cardiomyopathies: Results from a Dutch Multicenter Cohort Study. JACC Heart Fail.

[15] Lee SP, Ashley EA, Homburger J, Caleshu C, Green EM, Jacoby D (2018). Incident Atrial Fibrillation is Associated with MYH7 Sarcomeric Gene Variation in Hypertrophic Cardiomyopathy. Circ Heart Fail.

[16] Ho CY (2010). Genetics and Clinical Destiny: Improving Care in Hypertrophic Cardiomyopathy. Circulation.

[17] Marsiglia JD, Credidio FL, Oliveira TG, Reis RF, Antunes MO, Araujo AQ (2013). Screening of MYH7, MYBPC3, and TNNT2 Genes in Brazilian Patients with Hypertrophic Cardiomyopathy. Am Heart J.

[18] Mattos BP, Scolari FL, Torres MA, Simon L, Freitas VC, Giugliani R (2016). Prevalence and Phenotypic Expression of Mutations in the MYH7, MYBPC3 and TNNT2 Genes in Families with Hypertrophic Cardiomyopathy in the South of Brazil: A Cross-Sectional Study. Arq Bras Cardiol.

[19] Marsiglia JD, Credidio FL, Oliveira TG, Reis RF, Antunes MO, Araujo AQ (2014). Clinical Predictors of a Positive Genetic Test in Hypertrophic Cardiomyopathy in the Brazilian Population. BMC Cardiovasc Disord.

[20] The Share Registry (2024). Sarcomeric Human Cardiomyopathy Registry.

[21] Harris PA, Taylor R, Minor BL, Elliott V, Fernandez M, O'Neal L (2019). The REDCap Consortium: Building an International Community of Software Platform Partners. J Biomed Inform.

[22] Hespe S, Waddell A, Asatryan B, Owens E, Thaxton C, Adduru ML (2024). ClinGen Hereditary Cardiovascular Disease Gene Curation Expert Panel: Reappraisal of Genes associated with Hypertrophic Cardiomyopathy. medRxiv.

[23] Richards S, Aziz N, Bale S, Bick D, Das S, Gastier-Foster J (2015). Standards and Guidelines for the Interpretation of Sequence Variants: A Joint Consensus Recommendation of the American College of Medical Genetics and Genomics and the Association for Molecular Pathology. Genet Med.

[24] Ho CY, Day SM, Ashley EA, Michels M, Pereira AC, Jacoby D (2018). Genotype and Lifetime Burden of Disease in Hypertrophic Cardiomyopathy: Insights from the Sarcomeric Human Cardiomyopathy Registry (SHaRe). Circulation.

[25] Topriceanu CC, Pereira AC, Moon JC, Captur G, Ho CY (2024). Meta-Analysis of Penetrance and Systematic Review on Transition to Disease in Genetic Hypertrophic Cardiomyopathy. Circulation.

[26] Coppini R, Ho CY, Ashley E, Day S, Ferrantini C, Girolami F (2014). Clinical Phenotype and Outcome of Hypertrophic Cardiomyopathy Associated with Thin-Filament Gene Mutations. J Am Coll Cardiol.

[27] Moolman JC, Corfield VA, Posen B, Ngumbela K, Seidman C, Brink PA (1997). Sudden Death Due to Troponin T Mutations. J Am Coll Cardiol.

[28] Lopes LR, Syrris P, Guttmann OP, O'Mahony C, Tang HC, Dalageorgou C (2015). Novel Genotype-Phenotype Associations Demonstrated by High-Throughput Sequencing in Patients with Hypertrophic Cardiomyopathy. Heart.

[29] Wang S, Zou Y, Fu C, Xu X, Wang J, Song L (2008). Worse Prognosis with Gene Mutations of Beta-Myosin Heavy Chain than Myosin-Binding Protein C in Chinese Patients with Hypertrophic Cardiomyopathy. Clin Cardiol.

[30] Olivotto I, Girolami F, Ackerman MJ, Nistri S, Bos JM, Zachara E (2008). Myofilament Protein Gene Mutation Screening and Outcome of Patients with Hypertrophic Cardiomyopathy. Mayo Clin Proc.

[31] Ingles J, Yeates L, Semsarian C (2011). The Emerging Role of the Cardiac Genetic Counselor. Heart Rhythm.

[32] O'Mahony C, Jichi F, Pavlou M, Monserrat L, Anastasakis A, Rapezzi C (2014). A Novel Clinical Risk Prediction Model for Sudden Cardiac Death in Hypertrophic Cardiomyopathy (HCM risk-SCD). Eur Heart J.

[33] Monda E, Limongelli G (2023). Integrated Sudden Cardiac Death Risk Prediction Model For Patients with Hypertrophic Cardiomyopathy. Circulation.

[34] Marsili L, van Lint FHM, Russo F, van Spaendonck-Zwarts KY, Ader F, Bichon ML (2023). MYH7 p.(Arg1712Gln) is Pathogenic Founder Variant Causing Hypertrophic Cardiomyopathy with Overall Relatively Delayed Onset. Neth Heart J.

[35] Glazier AA, Thompson A, Day SM (2019). Allelic Imbalance and Haploinsufficiency in MYBPC3-Linked Hypertrophic Cardiomyopathy. Pflugers Arch.

[36] Flashman E, Redwood C, Moolman-Smook J, Watkins H (2004). Cardiac Myosin Binding Protein C: Its Role in Physiology and Disease. Circ Res.

[37] Daw EW, Chen SN, Czernuszewicz G, Lombardi R, Lu Y, Ma J (2007). Genome-Wide Mapping of Modifier Chromosomal Loci for Human Hypertrophic Cardiomyopathy. Hum Mol Genet.

[38] Tadros R, Zheng SL, Grace C, Jordà P, Francis C, Jurgens SJ (2023). Large Scale Genome-Wide Association Analyses Identify Novel Genetic Loci and Mechanisms in Hypertrophic Cardiomyopathy. medRxiv.

[39] Tavtigian SV, Harrison SM, Boucher KM, Biesecker LG (2020). Fitting a Naturally Scaled Point System to the ACMG/AMP Variant Classification Guidelines. Hum Mutat.

