# Supplementary material for: Caracterização Genética e Clínica de uma Coorte de Pacientes com Cardiomiopatia Hipertrófica no Sul do Brasil
Source: Arq Bras Cardiol. 2026 Mar 4;123(2):e20250420. [Article in Portuguese] doi: 10.36660/abc.20250420 (PMC13128179; doi:10.36660/abc.20250420)

Electronic Supplemental Material

Genetic and Clinical Characterization of a South-Brazilian Hypertrophic Cardiomyopathy Cohort

We present an interesting family case for detailed examination. As shown in **Figure 1S**, the pedigree includes the male index case (indicated by the arrow), who was initially diagnosed with a pathogenic MYH7 variant (mention here which one). Following this, the family underwent cardiologic evaluation and genetic screening using broader panels. The index patient's son was diagnosed with HCM and confirmed to have inherited the MYH7 pathogenic variant, along with a novel ALPK3 variant (mention here which one), which was later verified in the father's genetic test. The index patient's daughters did not exhibit any signs of the HCM phenotype, but both carried distinct ALPK3 variants - one classified as likely pathogenic, similar to those found in the rest of the family, and the other, a novel likely pathogenic truncating variant. Notably, both variants have not been previously reported in the literature. The mother was tested to determine the origin of the second novel variant but had a negative result. The son's daughter tested positive for both the MYH7 and the same ALPK3 variant as her father, though she showed no evidence of HCM at the time of evaluation (18 years old). Meanwhile, the daughter of the individual identified as a carrier of the mention here ALPK3 variant showed no signs of HCM until age 20, but confirmed inheritance of the same pathogenic variant.

Figure 1S. Hypertrophic Cardiomyopathy Family Pedigree.

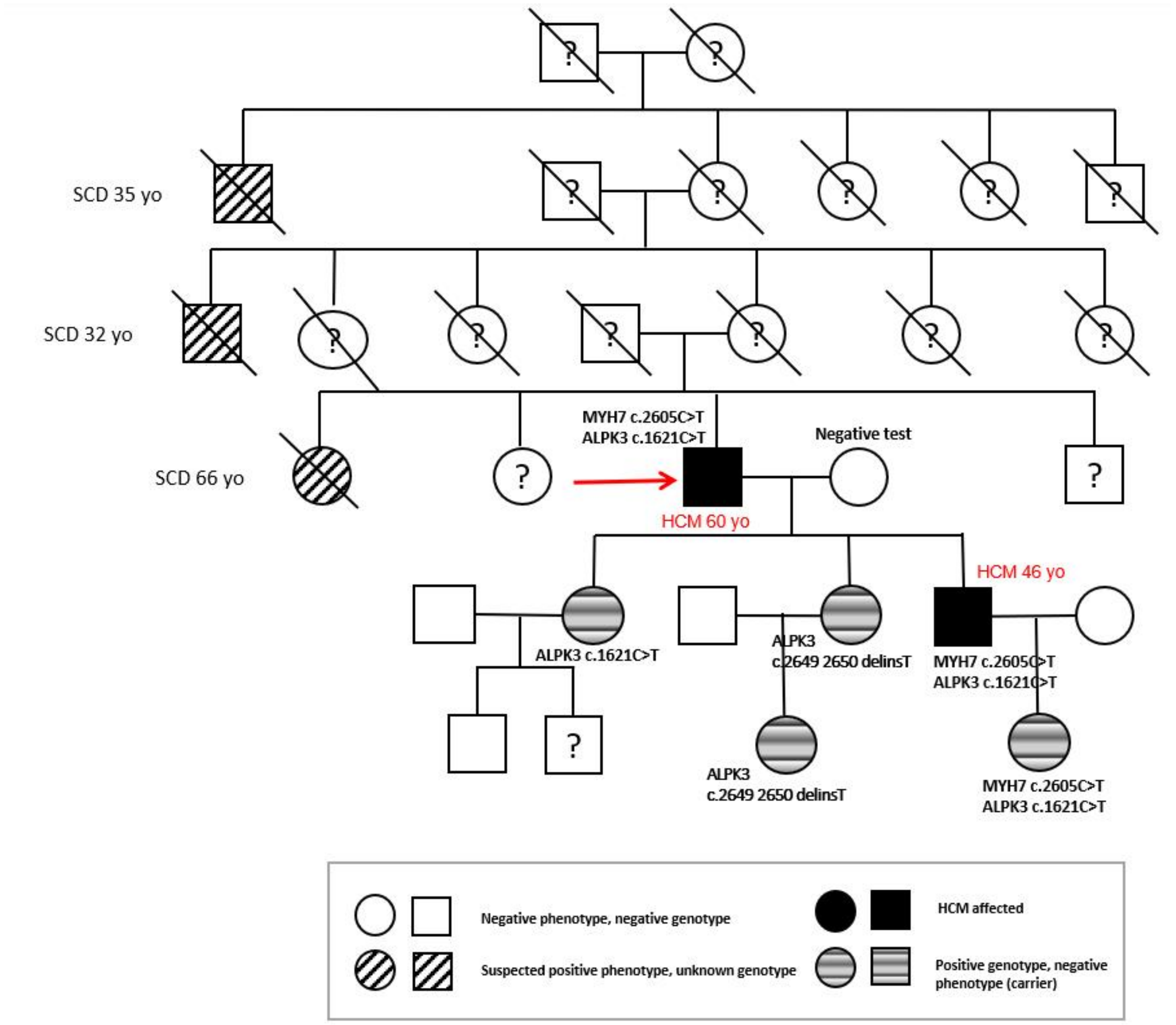

Supplement: Electronic Supplemental Material [file 0066-782x-abc-123-2-e20250420-suppl01.pdf]
