# Supplementary material for: Caracterização Genética e Clínica de uma Coorte de Pacientes com Cardiomiopatia Hipertrófica no Sul do Brasil
Source: Arq Bras Cardiol. 2026 Mar 4;123(2):e20250420. [Article in Portuguese] doi: 10.36660/abc.20250420 (PMC13128179; doi:10.36660/abc.20250420)
Supplement: Translation

# Translation: Genetic and Clinical Characterization of a South-Brazilian Hypertrophic Cardiomyopathy Cohort

**DOI:** 10.36660/abc.20250420i

**Authors:** Thais Beuren, Fernando Scolari, Fernanda Sperb-Ludwig, Filipe Ferrari, Arthur Rossi, Rafael Zawislak, Peter van Tintelen, Ricardo Stein

**Affiliations:** 1 Brazil Universidade Federal do Rio Grande do Sul, Porto Alegre, RS – Brazil; 2 Brazil Hospital Moinhos de Vento, Porto Alegre, RS – Brazil; 3 Brazil Hospital de Clínicas de Porto Alegre, Porto Alegre, RS – Brazil; 4 Brazil Pontifícia Universidade Católica do Rio Grande do Sul, Porto Alegre, RS – Brazil; 5 Netherlands University Medical Center Utrecht, Utrecht University, Utrecht – Netherlands

**Keywords:** Hypertrophic Cardiomyopathy, Genetic Testing, High-Throughput Nucleotide Sequencing, Brazil

## Abstract

**Background:**

Hypertrophic cardiomyopathy (HCM) is the most common monogenic heart disease, characterized by genetic and phenotypic heterogeneity. Although extensively studied in North American and European populations, data from Brazil remain limited.

**Objectives:**

To characterize the genetic and clinical profiles of a Southern Brazilian cohort of HCM patients and their relatives using massive parallel sequencing.

**Methods:**

In this observational study, HCM patients and first-degree relatives were recruited from outpatient cardiology clinics. Clinical and imaging data were collected, and genetic analysis used a 100-gene panel. Variant pathogenicity was assessed according to American College of Medical Genetics and Genomics criteria, and statistical analyses were performed using R software.

**Results:**

Eighty individuals were included in the final analysis (mean age: 49.2 ±18.5); 60% male; 40 index cases and 40 affected relatives). MYH7 and MYBPC3 were the most frequently related genes, with pathogenic / likely pathogenic variants (P/LP) identified in 33% and 16% of participants, respectively. No pathogenic TNNT2 variants were detected. Ninety percent of participants carried an identified variant (including variants of uncertain significance), with 68% harboring P/LP variants. MYH7 carriers exhibited a higher proportion of left ventricular outflow tract obstruction, whereas MYBPC3 carriers had a higher proportion of arrhythmic events and earlier diagnosis; however, these differences did not reach statistical significance and should be interpreted as exploratory. Clinical comparisons revealed regional differences, suggesting the potential impact of genetic diversity on the presentation of HCM in this part of Brazil.

**Conclusions:**

This study offers the first detailed genetic and clinical characterization of a Brazilian HCM cohort using massive parallel sequencing. Our findings underscore the importance of genetic testing for diagnosis, risk stratification, and management.

## Introduction

Hypertrophic Cardiomyopathy (HCM) is the most common monogenic heart disease, with a prevalence of approximately 1:250 to 1:500 individuals.^1^It is primarily caused by pathogenic variants in sarcomeric and sarcomere-related genes. The condition is characterized by left ventricular hypertrophy unrelated to abnormal loading conditions and may lead to complications such as Left Ventricular Outflow Tract Obstruction (LVOTO), Sudden Cardiac Death (SCD), and heart failure.^2 - 4^

Disease expression is highly variable, making the prediction of clinical outcomes challenging, even among genotype-positive individuals.^5 , 6^Certain gene variants have been associated with different prognoses: *MYH7* variants are linked to worse outcomes, while *MYBPC3* variants tend to present with delayed onset and incomplete penetrance.^7 - 10^Although genotype-specific risk prediction remains controversial, genotype-positive status itself has been associated with earlier disease onset, greater ventricular dysfunction, and increased cardiovascular mortality.^11 - 15^Accordingly, the latest AHA/ACC guidelines include genotype-positive status as a definitive risk factor for SCD in pediatric patients, with growing evidence in adults.^6^

Despite the recognized importance of genetics for diagnosis, risk assessment, and family counseling,^2 , 16^genetic evaluation remains underutilized in Brazil, and population-specific data are limited. Previous Brazilian studies have reported *MYH7* as the most prevalent mutated gene, followed by *MYBPC3* and *TNNT2* ,^17 - 19^a distribution consistent, though not identical to that observed in North American and European cohorts.^10 , 20^

This study aims to characterize the genetic and clinical profiles of patients with HCM and their relatives from a tertiary center in southern Brazil, using massive parallel sequencing.

## Methods

### Ethics statement

The study protocol was approved by the Institutional Ethics Committee through Plataforma Brasil (CAAE: 554413421.4.1001.5327).

### Participants and study design

The study employed an observational, cross-sectional design. Participants were recruited from the outpatient cardiology clinics of *Hospital de Clínicas de Porto Alegre* (HCPA), Brazil. Eligible individuals included patients diagnosed with HCM and their first-degree relatives.

Inclusion criteria for index patients required a confirmed diagnosis of HCM, defined as left ventricular wall thickness ≥ 15 mm not explained by abnormal loading conditions. For relatives carrying a Pathogenic (P) or Likely Pathogenic (LP) variant, inclusion in the study required genopositivity. Among enrolled relatives, a left ventricular wall thickness ≥ 13 mm was used to classify them as phenotype-positive (HCM), whereas asymptomatic carriers with wall thickness < 13 mm were considered phenotype-negative. Participants had to be 18 years or older at the time of enrollment. Exclusion criteria included the presence of HCM phenocopies, absence of clinical and genetic evidence of HCM, and age under 18 years. All participants received detailed information about the study and provided written informed consent before enrollment. Genetic counseling was offered to all participants both before and after testing.

### Database

The variables collected included date of birth, sex, and age at HCM diagnosis. Family history data were obtained, including a history of HCM and SCD, defined as at least one first- or second-degree relative who had experienced sudden, unexplained death or sudden cardiac arrest. Additional variables included episodes of aborted SCD and the presence of ventricular arrhythmias, such as sustained and non-sustained ventricular tachycardia or ventricular fibrillation. These arrhythmias were identified through 24-hour Holter monitoring, 12-lead resting Electrocardiogram (ECG), or exercise testing.

Other data collected included baseline pharmacological treatment, comorbidities, cardiac device implantation, presence of atrial fibrillation (detected via 24-hour Holter monitoring or 12-lead resting ECG) and mortality status. Electrocardiographic measures were also recorded. Echocardiographic parameters included Left Atrial (LA) size, Left Ventricular Ejection Fraction (LVEF), Left Ventricular End-Diastolic (LVED) and End-Systolic (LVES) diameters, left ventricular wall thickness, presence of Left Ventricle Outflow Tract Obstruction (LVOTO) and maximum outflow tract gradient. Cardiac magnetic resonance imaging parameters included LVEF, LVED and LVES volumes, left atrial volume and the presence of Late Gadolinium Enhancement (LGE).

### Genetic sequencing

Genomic DNA was extracted from saliva samples, and genetic testing was performed using a commercial comprehensive cardiomyopathy panel containing 100 genes: *ABCC9 ACADVL ACTC1 ACTN2 AGL ALMS1 ALPK3 BAG3 BRAF CACNA1C CACNA1D CALM1 CALM2 CALM3 CASQ2 CBL CDH2 CPT2 CRYAB CSRP3 DES DMD DNAJC19 DOLK DSC2 DSG2 DSP ELAC2 EMD EYA4 FHL1 FKRP FKTN FLNC, GAA, GATA4, GATA5, GJA5, GLA, HCN4, HRAS, JUP, KCNE1, KCNH2, KCNJ2, KCNQ1, KRAS, LAMP2, LMNA, LZTR1, MAP2K1, MAP2K2, MRAS, MTO1, MYBPC3, MYH6, MYH7, MYL2, MYL3, MYL4, MYLK3, NF1, NKX2-5, NRAS, PCCA, PCCB, PKP2, PLN, PPA2, PPCS, PPP1CB, PRKAG2, PTPN11, RAF1, RASA1, RBM20, RIT1, RYR2, SCN5A, SDHA, SGCD, SHOC2, SLC22A5, SOS1, SOS2, SPRED1, TAZ, TBX20, TCAP, TMEM43, TMEM70, TNNC1, TNNI3, TNNI3K, TNNT2, TPM1, TRDN, TRPM4, TTN, TTR, VCL* . Full-gene sequencing was performed using massive parallel sequencing technology.

### Statistical analysis

Study data were collected and managed using REDCap (Research Electronic Data Capture), a secure and confidential web-based platform.^21^Statistical analysis was performed using R software, version 4.3.0. Categorical variables were analyzed using chi-square tests. For continuous variables with normal distribution, independent t-tests were used, while variables without normal distribution were analyzed using the Wilcoxon Signed-Rank test. A significance threshold of 5% (p <0.05) was considered to all analyses.

## Results

### General Population Description

A total of 95 individuals, including affected patients and their relatives, were initially screened. Following genetic testing, relatives without a clinical diagnosis of HCM or an identified pathogenic or likely pathogenic variant were excluded (n=15). Consequently, 80 individuals were included in the final analysis: 40 unrelated index patients and 40 affected or variant-carrying relatives ( figure 1 ). Of these, 60% were men (n=48), 50% were index patients (n=40), and 50% were family members (n=40). Among the participants, 68% had an HCM diagnosis (n=54), while 32% were variant carriers (n=26). The mean (SD) age was 49.2 (18.5) years. A family history of HCM was presented in 32% of index patients (n=13).

**Figure 1:** – Selection process flowchart. Index: first identified case in the family; G+: genotype positive; G-: genotype negative; P+: phenotype positive; P-: phenotype negative.

The median left ventricular septal thickness was 18.5 mm (IQR: 15.0 - 21.0), the posterior wall thickness was 10 mm (IQR: 9.0 - 12.0), and the LVEF was 67% (IQR: 63.2 - 72.0). LVOTO was present in 33% of participants, and ventricular arrhythmias were observed in 15%. A summary of the clinical and demographic characteristics is provided in Table 1 .

**Table 1:** – Baseline characteristics of the patients

Characteristic	Total
**Gender - no. (%)**	
Female	32 (40)
Male	48 (60)
**Age (years)**	
Min	18
Max	86
Mean (SD)	49.2 ± 18.5
**Family history of HCM (index pts n=40) - no. (%)**	
Yes	13 (32.5)
No	27 (67.5)
**Family history of SCD - no. (%)**	
Yes	19 (25.6)
No	55 (74.4)
**Syncope - no. (%)**	
Yes	13 (17.3)
No	62 (82.7)
**Ventricular arrhythmia - no. (%)**	
Yes	12 (15)
No	68 (85)
**Comorbidities - no. (%)**	
Hypertension	20 (25.9)
Diabetes	10 (12.9)
Chronic Kidney Disease	3 (4)
Coronary Artery Disease	7 (9.3)
Dilated Cardiomyopathy	3 (4)
Implantable Cardioverter Defibrillator	10 (13.3)
Atrial Fibrillation	11 (14.6)

HCM: hypertrophic cardiomyopathy; SCD: sudden cardiac death.

### Genetic profile

Among all sequenced genes, P, LP, and Variants of Uncertain Significance were identified in 72 (90%) individuals. When considering only P and LP variants, 54 (68%) individuals were classified as having a positive genetic test. For initial characterization, all variants identified are shown in the Central Illustration . Figure 2 illustrates the distribution of P/LP variants in genes strongly or moderately associated with HCM.^22^The two most frequently found genes in HCM are highlighted: *MYH7* (33%) and *MYBPC3* (16%). Additionally, 10% of the sample had no identified variants.

**Figure 2:** – Distribution of affected genes strongly or moderately related to hypertrophic cardiomyopathy.

Six individuals exhibited HCM-related variants across more than one gene, with no specific pattern identified ( Table 2 ).

**Table 2:** – Individuals with multiple variants

ID	Genes	Variant	Pathogenicity	Affected	Family History of HCM	Age of diagnosis	LV wall	LVOTO	LV arrhythmia
1	MYH7	c.2605C>T	Pathogenic	Yes	Father	46	14mm	No	No
	ALPK3	c.1621C>T	Likely Pathogenic						
	TRPM4	c.1934G>A	VUS						
2	MYH7	c.1987C>T	Pathogenic	Yes	Index	23	17mm	No	No
	LZTR1	c.1957C>T	Likely Pathogenic						
3	MYBPC3	c.2047T>A	VUS* (~LP)	Yes	Index	47	17mm	No	Yes (NSVT)
	MYBPC3	c.3107G>A	VUS						
	RYR2	c.4273A>G	VUS						
4	ALPK3	c.2649 2650 delinsT	Pathogenic	No	Father	-	10mm	-	No
	TRPM4	c.1934G>A	VUS						
5	ALPK3	c.2649 2650 delinsT	Pathogenic	No	Grandfather	-	9mm	-	No
	DMD	c.5615C>T	VUS						
	GPD1L	c.1009G>C	VUS						
6	MYH7	c.2605C>T	Pathogenic	Yes	Index	60	20mm	Yes	No
	ALPK3	c.1621C>T	Likely Pathogenic						
	MYBPC3	c.292+138G>A	VUS						

HCM: hypertrophic cardiomyopathy; VUS: variant of unknown significance; LV: left ventricle; LVOTO: left-ventricular outflow tract obstruction. *VUS with high score in the American College of Medical Genetics and Genomics classification and potential for reclassification (Tavtigian et al.^39^).

To illustrate the importance of a comprehensive genetic and clinical evaluation, we present a detailed and intriguing family case (see Supplementary Material). Variant pathogenicity was classified according to the American College of Medical Genetics and Genomics (ACMG) criteria.^23^The distribution of variant pathogenicity classification was as follows: 1) pathogenic (44%) 2) likely pathogenic (34%), and 3) Variant of Unknown Significance (VUS, 22%). All variants identified in MYH7 and MYBP3 were missense, except for one intronic variant in MYH7. A detailed description of all variants can be found in Table 3 .

**Table 3:** – Variant description

Gene	Transcript	Variant	Classification	Population Frequency (GnomAD v.4.1.0)
ABCC9	NM_020297.4	c.3559G >T	VUS*	1.86e-6
ALPK3	NM_020778.5	c.1621C>T	LP	Not found
ALPK3	NM_020778.5	c.2649 2650 delinsT	P	Not found
CACNA1C	NM_000719.7	c.1342 G>A	VUS* (~LP)	5.59e-6
DMD	NM_004006.3	c.5615C>T	VUS	8.28e-7
DSG2	NM_001943.4	c.2505-2506insG	VUS	
DSP	NM_004415.4	c.1742C>T	VUS	Not found
FLNC	NM_001458.5	c.3964+1G>T	VUS*	Not found
GPD1L	NM_015141.4	c.1009G>C	VUS	1.86e-6
KCNH2	NM_000238.4	c.1898A>C	LP	Not found
KCNH2	NM_000238.4	c.526C>T	LB	3.27e-4
KCNH2	NM_000238.4	c.2863C>G	VUS	5.80e-5
LAMP2	NM_001122606.1	c.1234T>C	VUS	Not found
LZTR1	NM_006767.4	c.1957C>T	LP	2.48e-6
MYBPC3	NM_000256.3	c.1484G>A	P	2.42e-5
MYBPC3	NM_000256.3	c.1633C>A	VUS*	2.17e-5
MYBPC3	NM_000256.3	c.2047T>A	VUS*	Not found
MYBPC3	NM_000256.3	c.292+138G>A	VUS	
MYBPC3	NM_000256.3	c.3107G>A	VUS	7.09e-5
MYBPC3	NM_000256.3	c.3065G>C	VUS*	5.21e-5
MYBPC3	NM_000256.3	c.1505G>A	P	6.82e-6
MYH6	NM_002471.4	c.3010G>T	VUS	1.10e-3
MYH7	NM_000257.3	c.2737G>T	LP	
MYH7	NM_000257.3	c.4508G>A	P	
MYH7	NM_000257.4	c.1987C>T	P	1.86e-6
MYH7	NM_000257.4	c.2572C>G	P	2.48e-6
MYH7	NM_000257.4	c.2605C>T	P	1.86e-6
MYH7	NM_000257.4	c.3972+63A>CA	VUS	
MYH7	NM_000257.4	c.4402G>A	P	Not found
MYH7	NM_000257.4	c.1063G>A	P	4.34e-6
MYH7	NM_000257.4	c.2389G>A	P	2.85e-5
MYH7	NM_000257.4	c.788T>C	P	3.72e-6
MYL3	NM_000258.3	c.170C>G	P	5.14e-5
MYL3	NM_000258.3	c.187C>T	VUS	1.30e-5
PKP2	NM_001005242.3	c.2464A>G	VUS	3.97e-5
RYR2	NM_001035.3	c.1249C>G	VUS*	1.36e-5
RYR2	NM_001035.3	c.147G>T	VUS*	8.18e-6
RYR2	NM_001035.3	c.593A>T	VUS	1.86e-6
SCN5A	NM_000335.5	c.3612C>A	VUS	Not found
SLMAP	NM_001377540.1	c.2080C>T	VUS	6.20e-7
TRPM4	NM_017636.4	c.1934G>A	VUS	1.05e-5

LP: likely pathogenic; P: pathogenic; VUS: variant of uncertain significance; * VUS with high score in the American College of Medical Genetics and Genomics classification and potential for reclassification (Tavtigian et al.^39^)

### Clinical-genetic characterization of phenotype-positive individuals

To better understand how genetic background influences the HCM phenotype, we described and compared the clinical features of HCM patients with different affected genes ( Table 4 ). This analysis focused exclusively on genes with a well-established correlation to HCM, specifically *MYH7* and *MYBPC3* , based on the available sample size ( Table 5 ). Our comparison of *MYBPC3* and *MYH7* revealed noteworthy differences in frequencies, although these did not reach statistical significance. Patients with *MYH7* variants exhibited a higher frequency of LVOTO compared to those with *MYBPC3* variants (35% vs. 18%; p = 0.3). Conversely, patients with *MYBPC3* variants presented a more arrhythmogenic phenotype, characterized by a higher prevalence of ventricular arrhythmias (33% vs. 17%, p = 0.3), a higher rate of ICD implantation (27% vs 4%, p = 0.09), more frequent ventricular fibrosis, as identified by LGE on cardiac magnetic resonance (62% vs. 38%, p = 0.5), and a more frequent family history of sudden death (45% vs. 35%, p = 0.4). Additionally, the median age at diagnosis of *MYBPC3* -affected individuals was lower (37 years versus 46 years; p = 0.07). Other clinical characteristics are detailed in Table 5 .

**Table 4:** – Clinical characteristics of Genotype-Phenotype positive individuals

Characteristic	Total (N=53)	MYH7 (N=24)	MYBPC3 (N=12)	MYL3 (N=2)	ALPK3 (N=1)
**Gender - no. (%)**					
Male	33 (66)	16 (67)	7 (58)	2 (100)	1 (100)
Female	18 (34)	8 (33)	5 (42)	0 (0)	0 (0)
**Age - yr**					
Min	18	18	35	18	50
Max	86	86	73	32	50
Mean (SD)	54.9 ± 16.9	58.2 ± 17.3	51.1 ± 13.5	21.5 ± 14.8	50
**Age at HCM diagnosis - yr**					
Min	10	20	18	10	44
Max	73	73	60	29	44
Median (IQR)	44 (30.5–53.0)	46.5 (43.2–60.5)	37.5 (21.0–46.0)	19.5 (14.7–24.2)	44 (44.0–44.0)
**Left Ventricular Ejection Fraction - %**					
Min	30	30	63	75	68
Max	79	78	79	75	68
Median (IQR)	67 (63.2–72.0)	68.5 (64.0–72.0)	67 (64.5–70.5)	75 (75.0–75.0)	68 (68.0–68.0)
**Septum - mm**					
Min	7	10	10	13	14
Max	29	29	25	13	14
Median (IQR)	18.5 (15.0–21.0)	17 (13.2–21.0)	19 (17.5–21.0)	13 (13.0–13.0)	14 (14.0–14.0)
**Posterior Wall - mm**					
Min	7	8	8	9	9
Max	18	15	12	9	9
Median (IQR)	10 (9.0–12.0)	10 (9.0–12.0)	10 (9.0–10.5)	9 (9.0–9.0)	9 (9.0–9.0)
**Left Ventricular Outflow Tract Obstruction - no. (%)**					
No	32 (67)	13 (65)	9 (82)	1 (100)	1 (100)
Yes	16 (33)	7 (35)	2 (18)	0 (0)	0 (0)
**Ventricular Arrhythmia - no. (%)**					
No	41 (77)	20 (83)	8 (67)	2 (100)	1 (100)
Yes	12 (23)	4 (17)	4 (33)	0 (0)	0 (0)
**Atrial Fibrillation - no. (%)**					
No	40 (78)	14 (61)	9 (82)	1 (100)	1 (100)
Yes	11 (22)	9 (39)	2 (18)	0 (0)	0 (0)
**Late Gadolinium Enhancement - no. (%)**					
No	21 (60)	8 (62)	3 (37.5)	0 (0)	1 (100)
Yes	14 (40)	5 (38)	5 (62.5)	1 (100)	0 (0)
**Type of Ventricular Arrhythmia - no. (%)**					
NSVT	11 (21)	4 (17)	4 (33)	0 (0)	0 (0)
SVT	2 (4)	2 (8)	0 (0)	0 (0)	0 (0)
VF	1 (2)	0 (0)	0 (0)	0 (0)	0 (0)

SD: standard deviation; IQR: interquartile range; NA: non-applicable; LVOTO: left ventricle outflow tract obstruction; NSVT: non-sustained ventricular tachycardia; SVT: sustained ventricular tachycardia; VF: ventricular fibrillation

**Table 5:** – Clinical Characteristics of MYH7 and MYBPC3 Affected Individuals

Characteristic	MYH7 (N=24)	MYBPC3 (N=12)	p value
**Gender - no. (%)**			
Male	16 (67)	7 (58)	0.6003
Female	8 (33)	5 (42)	
**Age - yr**			
Min	18	35	
Max	86	73	
Mean (SD)	58.21 ± 17.33	51.17 ± 13.54	0.1925
**Age at HCM diagnosis - yr**			
Min	20	18	
Max	73	60	
Median (IQR)	46.5 (43.2–60.5)	37.5 (21.0–46.0)	0.0753
**Left Ventricular Ejection Fraction - %**			
Min	30	63	
Max	78	79	
Median (IQR)	68.5 (64.0–72.0)	67 (64.5–70.5)	1.000
**Septum - mm**			
Min	10	10	
Max	29	25	
Median (IQR)	17 (13.2–21.0)	19 (17.5–21.0)	0.319
**Posterior Wall - mm**			
Min	8	8	
Max	15	12	
Median (IQR)	10 (9.0–12.0)	10 (9.0–10.5)	0.4272
**LVOT Obstruction - no. (%)**			
No	13 (65)	9 (82)	0.3069
Yes	7 (35)	2 (18)	
**Ventricular Arrhythmia - no. (%)**			
No	20 (83)	8 (67)	0.3088
Yes	4 (17)	4 (33)	
**Atrial Fibrillation - no. (%)**			
No	14 (61)	9 (82)	0.2072
Yes	9 (39)	2 (18)	
**Late Gadolinium Enhancement - no. (%)**			
No	8 (62)	3 (37.5)	0.5344
Yes	5 (38)	5 (62.5)	
**Type of Ventricular Arrhythmia - no. (%)**			
NSVT	4 (17)	4 (33)	0.3088
SVT	2 (8)	0 (0)	0.8878
VF	0 (0)	0 (0)	NA
**Cardiac Devices - no. (%)**			
Implantable Cardioverter Defibrillator	4 (17)	3 (27)	0.094
Pacemaker	4 (17)	0 (0)	
ICD + PM	2 (9)	0 (0)	
CRT	0 (0)	0 (0)	
CRT + ICD	0 (0)	0 (0)	
None	16 (70)	8 (73)	
**Family history of SCD - no. (%)**			
No	3 (14)	1 (9)	0.567
Yes	8 (35)	5 (45)	NA
**Aborted SCD - no. (%)**			
No	22 (96)	12 (100)	1.000
Yes	1 (4)	0 (0)	

SD: standard deviation; IQR: interquartile range; ICD: implantable cardioverter defibrillator; ACP: artificial cardiac pacing; CRT: cardiac resynchronization therapy; NA: non-applicable; LVOTO: left ventricle outflow tract obstruction.

### Genotype-positive versus Genotype-negative affected individuals

Among individuals diagnosed with HCM, we compared characteristics of those with identified genetic variants (phenotype-positive/genotype-positive) to those with a negative genetic test (phenotype-positive/genotype-negative). The mean age at HCM diagnosis in genotype-positive individuals was 44 years, while it was 50 years (p = 0.86) in genotype-negative HCM patients. The median left ventricle septum thickness of genotype-positive individuals was 17.5 mm, compared to 21 mm of genotype-negative individuals (p = 0.18). Ventricular arrhythmias were present in 25% of genotype-positive and 12% of genotype-negative individuals (p = 0.77), atrial fibrillation in 23.3% and 12.5% (p = 0.83) and LGE in 48.2% and 0% (p = 0.08) of the genotype-positive and genotype-negative groups, respectively. A family history of SCD was reported in 38% of the genotype-positive group and 25% of the genotype-negative group (p = 0.76). Details are summarized in Table 6 .

**Table 6:** – Clinical Characteristics of Genotype + and Genotype - Affected Individuals

Characteristic	Negative (N=8)	Positive (N=45)	p value
**Gender – no. (%)**			0.8605
Male	6 (75)	29 (64)	
Female	2 (25)	16 (36)	
**Age – yr**			
Min	40	11	
Max	80	86	
Mean (SD)	58.12 ± 15.34	54.40 ± 17.29	0.5483
**Age at HCM diagnosis – yr**			
Min	50	10	
Max	50	73	
**Median (IQR)**	50 (50.0, 50.0)	44 (29.75, 53.50)	0.5631
Ejection Fraction – %			
Min	57	30	
Max	71	79	
**Median (IQR)**	66 (62.5, 67.5)	68 (64.0, 72.0)	0.1981
Septum – mm			
Min	7	10	
Max	26	29	
**Median (IQR)**	21 (18.7, 22.0)	17.4 (14.2, 21.0)	0.1884
Posterior Wall – mm			
Min	7	8	
Max	18	15	
**Median (IQR)**	11.5 (8.7, 13.5)	10.4 (9.0, 12.0)	0.4752
Left Ventricle Outflow Tract Obstruction – no. (%)			0.4936
No	4 (50)	28 (70)	
Yes	4 (50)	12 (30)	
**Ventricular Arrhythmia – no. (%)**			0.7753
No	7 (88)	34 (75)	
Yes	1 (12)	11 (25)	
**Atrial Fibrillation – no. (%)**			0.8328
No	7 (87.5)	33 (77)	
Yes	1 (12.5)	10 (23)	
**Late Gadolinium Enhancement – no. (%)**			0.082
No	6 (100)	15 (52)	
Yes	0 (0)	14 (48)	
**Type Ventricular Arrhythmia – no. (%)**			
Non Sustained Ventricular Tachycardia	1 (12.5)	10 (22)	0.8794
Sustained Ventricular Tachycardia	0 (0)	2 (4)	1
Ventricular Fibrillation	1 (12.5)	0 (0)	0.3249
**Cardiac Devices – no. (%)**			0.582
Implantable Cardioverter Defibrillator	2 (25)	6 (14)	
Artificial Cardiac Pacemaker	0 (0)	5 (12)	
ICD + ACP	0 (0)	3 (7)	
Cardiac Resynchronization Therapy	0 (0)	0 (0)	
CRT + ICD	0 (0)	0 (0)	
None	6 (75)	29 (67)	
**Family History of SCD – no. (%)**			0.7601
No	6 (75)	26 (62)	
Yes	2 (25)	16 (38)	

SD: standard deviation; IQR: interquartile range; ICD: implantable cardioverter defibrillator; CRT: cardiac resynchronization therapy; ACP: artificial cardiac pacing; LVOTO: left ventricle outflow tract obstruction.

## Discussion

In this study, we found that *MYH7* and *MYBPC3* were the most prevalent genes in the cohort of HCM patients from southern Brazil, with variants detected in 33% and 16% of the total individuals, respectively. These findings are consistent with previous studies, in which *MYH7* and *MYBPC3* were identified as the most common genes implicated in HCM, accounting for 30%-50% of cases.^2 , 3 , 10^However, our study reveals a slightly different distribution. Overall, the frequency of these variants aligns with what has been observed in other populations, although the proportion of cases without identified variants continues to pose a challenge for genetic diagnostics in HCM.

Interestingly, unlike most European and American cohorts, no individuals in our study were found to have variants in *TNNT2* .^24^It is important to note that around 30 genes have been implicated in HCM, with 21 of these showing moderate evidence for gene-disease association. To date, more than 2,000 variants have been documented.^9^Most of these encode sarcomeric proteins or are related to the sarcomere structure, which are critical components of the cardiac contractile machinery. Less common genetic variants, also linked to HCM, involve genes encoding sarcomere-related proteins such as *ACTN2, ALPK3, CSRP3, FHOD3, JPH2, KLHL24, MT-TI, PLN* and *TRIM63,* ^22^which in our cohort corresponded to a smaller proportion, as previously described. Observed differences in clinical features between *MYH7* and *MYBPC3* carriers, such as higher LVOTO in *MYH7* and trends toward arrhythmic events and earlier diagnosis in *MYBPC3* carriers, did not reach statistical significance. Therefore, these findings should be interpreted as exploratory and hypothesis-generating rather than indicative of causal relationships. Additionally, inclusion of multiple family members may introduce bias, as genetic and environmental factors shared within families could influence observed trends. Future studies with larger cohorts are needed to clarify potential genotype-phenotype correlations.

Unlike many previously reported cohorts, we found a higher proportion of P/LP variants. A meta-analysis by Topriceanu et al.^25^reports an overall P/LP variant rate of approximately 30-40% among HCM patients. Our findings could reflect selection bias due to the limited availability of genetic tests, with a preference for selecting patients with a higher likelihood of a positive test (i.e., familial cases). Additionally, individuals with more frequent cardiology visits – often those with more severe phenotypes – may have been more likely to undergo genetic testing.

This first report on a Brazilian HCM cohort with extensive genetic screening aims to clarify genotypic findings, including those related to less-established genes. However, no specific patterns were found, despite some individuals carrying multiple variants. The distribution of variants may differ across generations, and it remains unclear how disease severity is influenced by a single variant compared to multiple variants. Previous reports suggest that modifier genes or additional sarcomeric gene variants may contribute to a worse phenotypic expression of HCM.^14 , 26 , 31^In our cohort, we observed two VUS in genes with limited evidence for association with HCM, namely *ABCC9* and *RYR2* . Furthermore, we identified a young female football player who exhibited significant left ventricular hypertrophy without fulfilling the criteria for Noonan syndrome. She carried a pathogenic *MYH7* variant and a pathogenic *LZTR1* variant (associated with Noonan syndrome), which may represent a phenocopy. An intriguing example is also provided by a family in which four individuals carried pathogenic variants in both *MYH7* and *ALPK3* , as detailed in the Supplementary Material.

It is important to emphasize the impact of diagnosing genetic heart diseases, not only on patients but also on at-risk family members. Genetic counseling is indispensable, as it has been shown to reduce anxiety, improve risk perception, enhance disease knowledge, and promote emotional support.^31^Additionally, multidisciplinary case discussions are invaluable and essential for the effective management of these patients.

Linking the genetic profile to disease expression in HCM – whether in terms of phenotype or adverse outcomes – remains a major challenge. Current risk prediction tools for SCD primarily rely on established clinical markers such as left ventricular wall thickness, syncope, left ventricular systolic dysfunction, LVOTO, non-sustained ventricular tachycardia, LGE, and family history of SCD.^32 , 33^Previous studies have reported inconsistent results regarding genotype-phenotype correlations in disease severity. Watkins et al.^13^demonstrated reduced life expectancy in patients with *MYH7* missense variants, while Jansen et al.^14^similarly observed poorer clinical outcomes associated with *MYH7* mutations. In addition, *MYH7* variants have been linked to elevated atrial fibrillation risk.^15^However, emerging evidence suggests certain *MYH7* variants may correlate with attenuated disease manifestations. Notably, Marsili et al.^34^recently identified delayed disease onset with gradual symptom progression, particularly among female carriers of the *MYH7* p.Arg1712Gln pathogenic variant. These contrasting findings underscore the importance of more nuanced investigations into genotype-phenotype relationships, accounting for variant-specific effects.

Meanwhile, pathogenic *MYBPC3* variants have been linked with later disease onset and higher rates of incomplete penetrance compared to *MYH7* variants.^14^Conversely, certain *TNNT2* variants (Arg92Trp, Arg92Gln, and Ile79Asn) have been associated to increased SCD risk in affected families.^27^Although differences among affected individuals in our cohort did not reach statistical significance, it is plausible that patients with positive genotypes experience more severe disease. Importantly, in our study, carriers of *MYBPC3* variants showed a higher proportion of features associated with SCD – including more ventricular arrhythmias, higher rates of ICD implantation, greater prevalence of family history of SCD, and earlier age of HCM diagnosis.

On the other hand, we observed greater LVOTO frequency among *MYH7* variant carriers, while the prevalence of heart failure appeared similar across groups. While *MYH7* mutations disrupt sarcomeric protein interactions, *MYBPC3* pathogenicity seems primarily driven by haploinsufficiency – either from nonsense-mediated mRNA decay or the production of defective proteins unable to integrate into the sarcomere.^35 , 36^A Brazilian study by Marsiglia et al. supports this hypothesis by identifying a higher proportion of truncating *MYBPC3* variants.^17^In our analysis, all *MYBPC3* variants were missense; however, this does not necessarily imply a milder phenotype.

Collectively, these findings highlight the need for further investigation into the molecular pathogenic mechanism of *MYBPC3* . Despite numerous studies, no definitive genotype-phenotype predictors have been established. Disease expression likely varies by variant, rather than by gene alone. Besides traditional cardiovascular risk factors (e.g., hypertension, overweight) influencing HCM development, common genetic variants have been shown to account for a significant portion of disease heritability.^37 , 38^Thus, detailed characterizing specific variants may help refine polygenic risk scores in the future. Ongoing global efforts are likely to drive progress in this area.

Although the relationship between specific genes and disease severity remains controversial, the presence of a positive genotype (regardless of gene) had consistently been associated with worse clinical outcomes. Ho et al.^16^identified positive genetic testing as the best independent predictor of adverse events in HCM patients. Similarly, Marsiglia et al.^19^observed that Brazilian individuals with positive genotypes had earlier HCM diagnosis and higher ventricular arrhythmia incidence. In our study, there was no difference between groups, even though we observed similar proportions: a higher frequency of ventricular arrhythmias, atrial fibrillation, and left ventricle fibrosis in the genotype-positive patients, along with earlier diagnosis and more positive family histories of SCD. These findings reinforce the importance of recognizing HCM as a genetically driven disease, irrespective of the specific gene involved.

Despite the inherent complexity of HCM, there is substantial evidence supporting the central role of genetics in disease management. The autosomal dominant inheritance pattern, with a 50% chance of transmission, has major implications for the medical care and life decisions of at-risk relatives.^1^In Brazil, as in many developing countries, genetics is often underutilized in HCM management due to high cost, limited physician knowledge, and insufficient awareness of its clinical impact. This results in suboptimal patients care. To improve HCM management, it is crucial to promote more comprehensive genetic testing using advanced genomic techniques in larger population samples. We believe that advocating for the integration of genetics into routine HCM management could gradually lower costs and increase access, ultimately improving care for HCM patients in developing countries.

### Limitations

The major limitation of our study is the small, locally based sample size. Given the heterogeneity of HCM, a larger cohort including individuals from diverse regions of Brazil would provide a more comprehensive understanding of the genetic characteristics of our population. Moreover, the inclusion of patients primarily from a tertiary center may have introduced selection bias, as these individuals are more likely to represent a severe or specialized subset of HCM cases. This may have influenced both the frequency and classification of the variants identified.

Besides, the inclusion of family members might have inflated the diagnostic yield of genetic testing. The exclusion of phenotype-negative relatives without pathogenic variants also limits our ability to estimate penetrance and assess genotype-phenotype correlations across families. Formal analyses of familial aggregation and genetic segregation were not performed due to the limited sample size and cross-sectional design. In addition, this study did not assess the clinical impact of genetic testing, such as potential changes in patient management or family screening practice, nor did it include longitudinal follow-up to assess clinical outcomes such as sudden death, hospitalization, or progression to heart failure **.** Expanding the sample to include patients from primary and secondary care centers could help mitigate this bias and provide a more accurate representation of the broader Brazilian HCM population.

## Conclusion

In this cohort of southern Brazilian HCM patients and their relatives, *MYH7* and *MYBPC3* were the most frequently mutated genes, with no pathogenic variants identified in *TNNT2* . We observed a higher frequency of P/LP variants compared to international cohorts. Hypothesis on the non-significant associations of MYBPC3 variants with markers of disease severity should be explored in further studies. These findings suggest important genetic and phenotypic heterogeneity within this Brazilian HCM cohort and reinforce the clinical value of genetic testing for risk stratification and management.

## Supplemental Materials

Electronic Supplemental Material
